# Self-reported visual symptoms and high visual demand activities in professional football players: a cross-sectional survey

**DOI:** 10.3389/fspor.2023.1256847

**Published:** 2023-12-14

**Authors:** Jorge Jorge, José Teixeira, Tiago Pinhão, Frederico Delgado, Alshaarawi Salem, Francesco Martino, Sotiris Plainis

**Affiliations:** ^1^ Clinical and Experimental Optometry Research Laboratory (CEORLab), Physics Center of Minho and Porto Universities (CF-UM-UP), School of Sciences, University of Minho, Braga, Portugal; ^2^ Medical Department, Rio Ave Futebol Clube, Vila do Conde, Portugal; ^3^ TP - Terapias Partilhadas, Braga, Portugal; ^4^ Fisio Go – Clinic, Maia, Portugal; ^5^ Department of Physics, University of Minho, Braga, Portugal; ^6^ Optics Department, Granada University, Granada, Spain; ^7^ Laboratory of Optics & Vision, School of Medicine, University of Crete, Heraklion, Greece

**Keywords:** convergence insufficiency, football (soccer), smartphone, sports vision, binocular vision

## Abstract

**Background:**

Vision is crucial for football players, impacting decision-making and athletic performance. Despite its global popularity, football lacks comprehensive evaluations of the impact of digital device use on ocular symptoms during high-demand activities.

**Purpose:**

To gain knowledge about the time spent by football players in high visual demand activities, the symptoms associated with binocular vision dysfunction, and their relationship with sports performance.

**Methods:**

A cross-sectional observational study was conducted in 2020 using an online survey targeting football players from Portugal, England, Spain, and Saudi Arabia. The survey, distributed over 5 weeks, aimed to collect data from approximately 5,000 football players. Information on player profiles, competitive levels, vision habits, and symptoms related to binocular vision dysfunctions was collected. The Convergence Insufficiency Symptom Survey (CISS) employed a 5-point Likert scale to indicate the average frequency of each symptom. Due to non-normality, non-parametric tests were used (*p* < 0.05). Specifically, Mann-Whitney U, Kruskal-Wallis, Chi-square, and Spearman's rank correlation tests were used as appropriate.

**Results:**

Analyzing male professional football players (mean age: 27.4 ± 5.0 years, 95% CI, 26.7–28.1), it was found that 38.1% of the players had been called up to the national team and 6.9% had played over 50 games. Self-rated last season's performance had a mean score of 6.5 ± 2.1 (95% CI, 6.2–6.8)(on a scale of 1 to 10). Smartphone use exceeded 1 h daily for all players, with 36.0% surpassing 4 h. Visual symptoms, notably associated with smartphone use (35.5%), were observed. Regarding the CISS score, the mean was 7.1 ± 7.7 (IC95%: 6.6 to 8.8). A weak negative correlation (rho = −0.215, *p* = 0.003) emerged between CISS scores and self-perceived sports performance. Football players using prescription lenses had significantly higher CISS scores (11.9 ± 10.4, 95% CI, 12.3–7.7) compared to non-users (6.2 ± 6.8, 95% CI, 7.8–5.7) (*p* < 0.001).

**Conclusion:**

This study reveals that professional football players engage in high visual demand tasks, notably on smartphones. One-third of the players link smartphone use to ocular symptoms. The Convergence Insufficiency Symptoms Survey indicates that 6.3% exhibit binocular vision dysfunction symptoms. Those with fewer ocular symptoms perceive that they have better sports performance than their counterparts.

## Introduction

Vision affects daily activities such as walking, reading, or practicing sports, and when the visual system malfunctions or is exposed to visually demanding tasks, it affects not only ocular health but also musculoskeletal health. Extensive research highlights the importance of vision in various sports, particularly in football, where players continuously process visual information for decision-making. Beyond visual acuity and refractive error, binocular vision plays a critical role in accurately assessing distances and positions in the player's surroundings. The strong link between binocular vision, especially stereopsis, and athletic performance is evident, considering the precision required for spatial judgment in football. Stereopsis significantly enhances visual search and decision-making processes, making effective binocular vision, influenced by various factors, a prerequisite for optimal athletic performance (1–7). Research in football has unveiled the diverse visual abilities required for different positions on the field. Furthermore, a recent investigation revealed distinct visual search strategies employed by athletes based on their positions. The findings indicate that central midfielders and central defenders rely heavily on scanning, while forwards utilize it less frequently (5, 8, 1–7). Despite the studies carried out and the growing interest in the relationship between vision and football, there are still areas that need to be studied in more depth. For example, it is important to better understand the influence of visual dysfunctions on sports performance. It is also necessary to investigate the repercussions of visual habits on players' vision. In addition, it is important to establish accurate and rigorous routines for visual assessment and training for each sport. Football players, by the characteristics of their profession, have daily and weekly cycles of training and competition variables (9, 10). In these cycles, part of their time is used in high visual demand activities such as playing video games, watching television, or using the smartphone. Football players report that they use smartphones, television sets, or computers for periods ranging from 30 min to several hours, including, as a strategy to fall asleep (11). The use of these devices in the adult population has been associated with an increase in symptoms and the appearance of binocular vision and accommodative dysfunctions. To date, countless studies mention the influence of these kinds of devices and time of use on visual dysfunctions (12–15).

As observed in the general population, it is to be expected that football players exposed daily to high visual demand activities can also develop binocular vision dysfunction symptoms and that these symptoms can have an impact on their daily activities. It is therefore important to study the vision habits of football players and to understand the symptoms that may be associated with such habits. There are several surveys to highlight the symptoms of binocular vision dysfunction, with one of the most used being the Convergence Insufficiency Symptom Survey (CISS), which has been translated into several languages (16–19). The new version of this survey (15-item version) is a frequently used outcome measure in binocular vision research and has been used to assess convergence insufficiency symptoms in several clinical groups of different ages (20, 21). Several publications state that the Convergence Insufficiency Symptom Survey is not a condition-specific instrument for convergence insufficiency and it could be useful for measuring the symptoms associated with visual discomfort caused by other factors.(17, 19).

In the current literature, apart from studies linking concussions and visual symptoms (22, 23), there is a dearth of scientific publications connecting the symptoms of visual dysfunctions with football practice. In the clinical and sports context, some surveys are used to assess the visual status of football players. However, these surveys focus only on the football player's ocular history and the use of glasses or contact lenses, neglecting the relationship between vision and sports performance. They also completely ignore the football player's visual habits and the symptoms associated with them (24).

The present study hypothesizes that professional football players engage extensively in high visual demand activities, exhibit a lower prevalence of symptoms associated with vision dysfunctions compared to the general population, and demonstrate a positive correlation between reported symptoms, sports performance, and the time allocated to high visual demand activities. This study investigates the complex interplay between vision dysfunctions, high visual demand activities, and sports performance in professional football players, with a specific emphasis on the potential adverse effects of smartphone usage. The findings have broad implications for football players, coaches, sports medicine professionals, and vision specialists, highlighting the imperative for collaborative efforts to safeguard football players’ visual health and optimize their overall athletic performance.

The objectives of this study were to quantify the duration of engagement in high visual demand activities in professional football players, assess symptoms associated with vision dysfunctions, and investigate the relationship between reported symptoms, sports performance, and the time allocated to such demanding visual activities.

## Methods

A cross-sectional observational study was conducted using an online survey methodology, which was released to several football clubs in the second half of 2020 over a period of 5 weeks; the collaboration of football players was requested for them to complete the survey. The survey was distributed in Portugal, England, Spain, and Saudi Arabia, corresponding to a potential universe of approximately 5,000 professional football players. This led to the need for a sample size of 235 participants (25). The online survey (hosted by Google Forms) required 10 min to be completed and was divided into three parts: the first part collected demographic data about the football player and his competitive level; the second part collected data on vision habits and ocular history; and the third part focused on the symptoms related to binocular vision dysfunctions at near vision.

The questions in the first part were: age, sex, race/ethnicity, nationality, professional activity in addition to football, competitive level, country where the player works/plays, division where he plays, and his position on the field.

The questions aimed at assessing the player's performance and competitive level were as follows:

1) “In the last season, what percentage of games have you played?” with response options being less than 10%, between 10% and 25%, between 25% and 50%, between 50% and 75%, more than 75%, and does not apply in my case.
2) “How do you rate your performance in the current sports season?” with players having to choose on a scale from 1 to 10, where 1 corresponds to weak and 10 to great.
3) “If the performance has been lower than expected, is there any reason for the decrease in performance?” with response options of no, technical option, lesion, and other options.
4) “Have you been called up for the national team?” with response options being never, less than 10 times, less than 20 times, less than 50 times, more than 50 times, and does not apply to my case.

These questions provided valuable insights into the player's engagement, self-perceived performance, potential reasons for performance decline, and national team involvement. The questions in the second part of the survey were: 1) “Have you ever been submitted to any: Visual Exam? If yes, how long?; Eye surgery? If yes, how long?; Vision training? If yes, how long?”. 2) “Have you ever suffered from An eye Injury? If yes, how long?; Cerebral concussion? If yes, how long?”.

Regarding the use of visual correction, the questions were: “Do you wear glasses or contact lenses for day-to-day activities?”, “Do you wear contact lenses to play?”, and “Do you wear contact lenses for training?”. The questions regarding vision habits were: “On average, how many hours a day do you spend on the following activities: smartphone; tablet; computer; game consoles; television; reading printed material; outdoor/sports activities.” Symptoms related to binocular vision dysfunction at high visual demands were assessed with the revised 15-item Convergence Insufficiency Symptom Survey (CISS) in the third part of the survey. The frequency of each symptom was rated on a 5-point Likert scale (0 = Never, 1 = Not often, 2 = Occasional, 3 = Fairly Often, and 4 = Always), with the sum of the items' scores used to give a total score out of 60. The current literature states that the CISS is a valid and repeatable survey for measuring symptoms associated with visual dysfunctions, particularly those related to binocular vision. It also indicates that score values ≥21 serve as an effective cutoff point in differentiating between symptomatic and asymptomatic individuals (26–28). Regarding the association of symptom appearance or increase in symptoms with specific activities, the question was: “Do you associate the appearance or increase of any of the symptoms in the previous question with any specific activity?” with the response options being: none, smartphone, tablet, computer, game consoles, television, reading printed material, and outdoor/sports activities. The study was conducted in accordance with the Declaration of Helsinki and was approved by the Ethics Subcommittee for Life and Health Sciences of the University of Minho.

All data were analyzed using IBM SPSS Statistics version 27(IBM Inc). The normality of data was assessed using the Kolmogorov-Smirnov test. All variables studied were non-normally distributed (*p* < 0.001, Kolmogorov-Smirnov test). Therefore, non-parametric tests were used for all analyses. Differences were considered statistically significant when the *p*-value was lower than 0.05. Specifically, Mann-Whitney U, Kruskal-Wallis, Chi-square, and Spearman's rank correlation tests were used as appropriate.

## Results

A total of 189 professional football players participated in the study, representing a subsample of the 255 participants who completed the survey. Only professional football players with no other regular professional activities were included in the final analysis. Tables 1–3 present the most relevant survey parameters for professional football players. Table 1 outlines the participants' demographics and football-related characteristics. Table 2 summarizes the time spent on various daily activities and its correlation with symptom onset. Table 3 presents the data obtained from the CISS survey.

**Table 1:** Demographics and football-related questionnaire data for all the sample.

Age (Years)^a^		27.4 ± 5.0 (95% CI 26.7–28.1)
Ethnicity	African	33 (17.5%)
	Asian	2 (1.1%)
	Arabian	31 (16.4%)
	Caucasian	108 (57.1%)
	Latin-American	14 (7.4% )
	Mixed	1 (0,5%)
Position	Goalkeeper:	28 (14.8%)
	Defender:	69 (36.5%)
	Midfielder:	54 (28.6%)
	Forward:	38 (20.1%)
Championship level	1st league	113 (59.8%)
	2nd league	39 (20.6%)
	3rd league	37 (19.6%)
Percentage of games played in the last two seasons	<10%	9.5%
	10% to 25%	6.3%
	25% to 50%	8.5%
	50% to 75%	32.3%
	≥75%	43.4%
Calls-up to the national team	Never	117 (61.9%)
	<10 times	36 (19.0%)
	10 to 20 times	11 (5.8%)
	21 to 50 times	12 (6.3%)
	>50 times	13 (6.9%)
Last season performance (self-perception) (scale 1 to 10)^a^		6,5 ± 2.1 (95% CI, 6.2–6.8)

^a^ mean and standard deviation.

**Table 2:** Displays data from the survey on daily life activities and their relationship with the onset of symptoms for the entire sample. The values representing the relationship between symptom onset and specific activities exceeded 100%, indicating that certain football players attribute the onset of symptoms to more than one activity.

Hours per day spent on	M ± IQ^a^	95% CI
Smartphone	3 ± 2	1.9–2.6
Tablet	0 ± 1	0.3–1.0
Computer	0 ± 1	1.0–2.4
Game consoles	0 ± 1	1.2–2.2
Television	2 ± 2	1.6–2.6
Reading printed material	1 ± 1	0.3–0.6
Outdoor/sports activities	2 ± 1	1.2–1.9
Activity related to the symptom´s onset	Percentage	
None	48.7%	
Smartphone	35.5%	
Tablet	3.2%	
Computer	8.5%	
Game consoles	14.8%	
Television	14.3%	
Reading printed material	8.5%	
Outdoor/sports activities	1.6%	

^a^ median and interquartile range.

**Table 3:** CISS data for all the sample.

	Question	M ± IQ^a^	95% CI
#1	Do your eyes feel tired when reading or doing close work?	1 ± 2	0.7–1.1
#2	Do your eyes feel uncomfortable when reading or doing close work?	0 ± 1	0.5–1.0
#3	Do you have headaches when reading or doing close work?	0 ± 1	0.5–0.9
#4	Do you feel sleepy when reading or doing close work?	0 ± 1	0.6–1.1
#5	Do you lose concentration when reading or doing close work?	0 ± 1	0.5–0.9
#6	Do you have trouble remembering what you have read?	0 ± 1	0.6–1.0
#7	Do you have double vision when reading or doing close work?	0 ± 0	0.3–0.7
#8	Do you see the words move, jump, swim, or appear to float on the page when reading or doing close work?	0 ± 0	0.1–0.4
#9	Do you feel like you read slowly?	0 ± 1	0.4–0.6
#10	Do your eyes ever hurt when reading or doing close work?	0 ± 1	0.4–0.8
#11	Do your eyes ever feel sore when reading or doing close work?	0 ± 0	0.3–0.6
#12	Do you feel a “pulling” feeling around your eyes when reading or doing close work?	0 ± 0	0.3–0.7
#13	Do you notice the words blurring or coming in and out of focus when reading or doing close work?	0 ± 0	0.2–0.6
#14	Do you lose your place while reading or doing close work?	0 ± 0	0.3–0.6
#15	Do you have to re-read the same line of words when reading?	0 ± 1	0.6–1.0

^a^ median ± interquartile range.

All participants were men and the mean age was 27.4 ± 5.0 years [range: 18–39 years; 95% confidence interval (CI): 26.7–28.1 years]. The majority of participants were Caucasian (57.1%). Approximately 59.5% of the participants played in the first division. In terms of sports performance, Only 38.1% of the participants were called up to the national team at least once, and only 6.9% played more than 50 games for the national team.

Participants rated their own performance in the last season using a scale of 1 to 10, where 1 meant very bad and 10 meant very good. The mean of responses was 6.5 ± 2.1 (range: 2–10; 95% CI: 6.2–6.8). The most common ratings were 8 (29.1%) and 7 (24.9%). Only 3.2% of participants rated the season with 10, and 5.3% rated it with 1. The main causes pointed out by the players for a decrease in performance during the season were technical issues (25.4% of responses) and injuries (19.6%).

Approximately 16.4% of participants wear glasses or contact lenses for everyday tasks. However, only 5.8% wear contact lenses for playing or training.

All football players reported spending at least 1 h per day using their smartphones, with 36.0% reporting using them for 4 or more hours per day. Regarding the use of tablets, computers, and game consoles, respectively, 71.4%, 60.8%, and 55.0% of football players reported not engaging in these activities.

**Figure 1:**
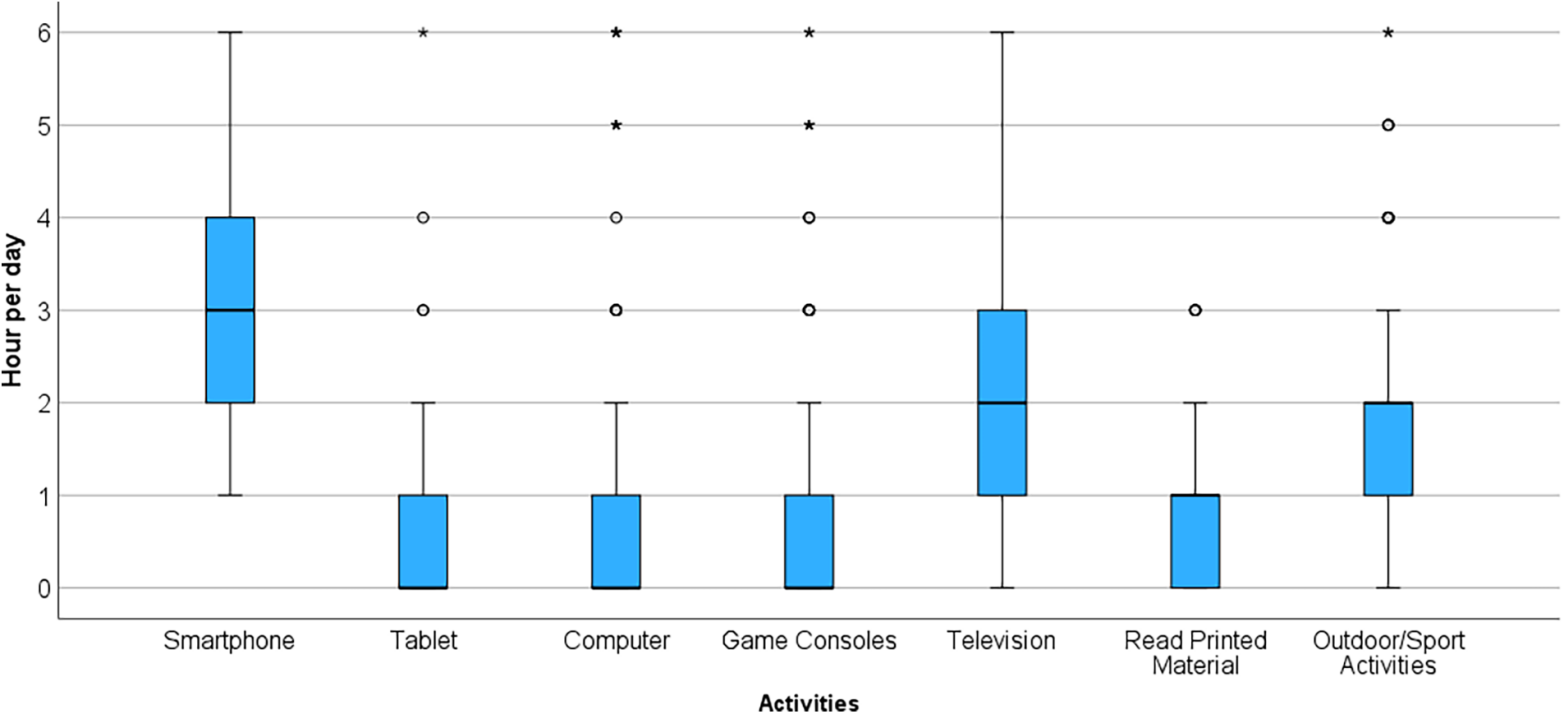
Graphical representation of time spent by football players in different daily activities.

As illustrated in Figure 1, football players allocated more daily time to smartphone use than to any other activity, followed by television viewing. The remaining activities, including outdoor activities, had a residual usage pattern, indicating that players engaged in these activities for a relatively short period. The median daily time spent on outdoor activities was 2 ± 1 h (95% CI, 1.2–1.9 h), suggesting that football players dedicate little time to outdoor activities beyond their training-related activities.

Among the reported symptoms in the CISS, 51.3% of football players associated them with at least one daily activity, with smartphone use being the most commonly associated activity (35.5%).

**Figure 2:**
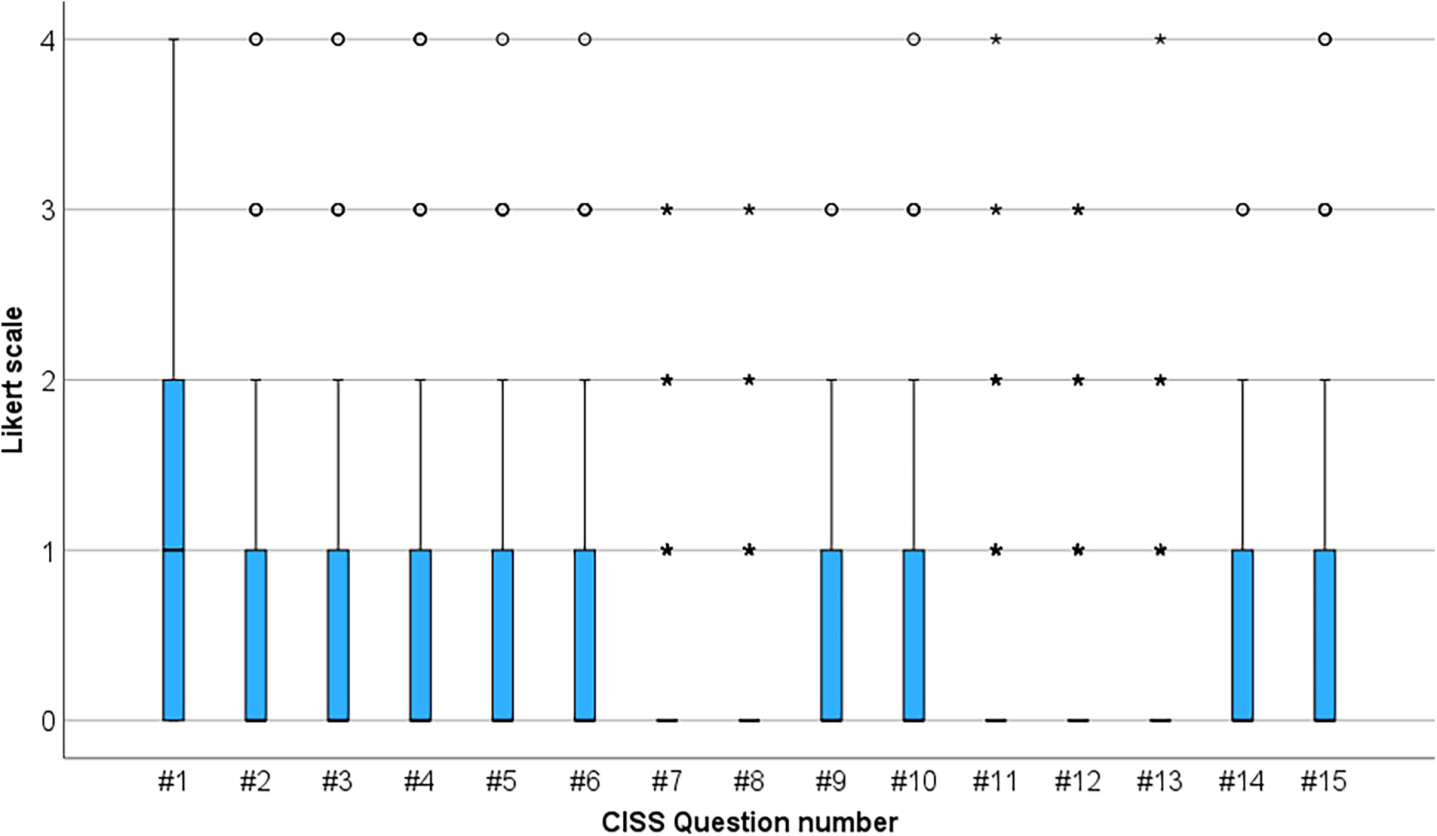
Graphical representation of symptoms reported in the CISS Survey among professional football players.

A mean CISS score of 7.1 ± 7.7 (95% CI: 6.6–8.8) was obtained. Furthermore, 6.3% of the football players had a CISS score of 21 or higher. Questions #1, #15, #2, #4, and #6, in that order, were the ones with the highest frequency of symptom reporting among the football players. Specifically, 26.5%, 21.2%, 17.5%, 16.9%, and 15.3% of the football players reported “occasionally,” “very often,” or “always” experiencing the symptoms associated with questions #1, #15, #2, #4, and #6, respectively. A small number of players reported visual symptoms related to questions #7, #8, #11, #12, and #13 (Figure 2).

The CISS score was analyzed for correlations with various parameters obtained from the general survey. Statistically significant correlations were identified using Spearman's rho test. A negative correlation was found between self-perception of sports performance in the last season and the CISS score (Spearman's rho = −0.215, *p* = 0.003). However, despite statistical significance, the coefficient of −0.215 indicates a weak association between a football player's perception of their sporting performance and the symptoms reported in the CISS score. This suggests that an improved perception of performance corresponds to fewer reported symptoms.

Furthermore, football players who used glasses or contact lenses in their daily and sports activities had a higher CISS score than those without any corrective eyewear. Users of corrective eyewear had a mean CISS score of 11.9 ± 10.4 (95% CI 8.1–15.7), while non-users had a mean score of 6.2 ± 6.8 (95% CI 5.1–7.2). This difference was statistically significant (*p* < 0.001).

## Discussion

A recent review by Laby et al. on sports vision research found that despite numerous studies on visual assessment and training in athletes, few have directly linked these capabilities to on-field performance in competitive matches. Studies have been conducted in various sports, with baseball, basketball, marksmanship, and hockey being the most studied. Results indicated that in baseball, ocular dominance, stereoacuity, and eye-hand coordination showed no significant associations with batting or pitching statistics across diverse player samples. Additionally, studies revealed significant correlations between the ability to rapidly identify pitch images and batting averages, as well as between accuracy in judging pitch types and batting performance. In marksmanship, elite shooters exhibited earlier and longer durations of the “quiet eye,” particularly in successful trials. These findings underscore the intricate dynamics of the associations between visual abilities and athletic success (29).

The purpose of the study was to understand the vision habits of football players and the symptoms associated with daily activities. An online survey was used, which in addition to the usual demographic characteristics, also included the CISS, which had been previously validated to study the symptoms associated with binocular vision dysfunctions (16–18).

The mean of the sum of the responses from the CISS was lower than those currently published for the adult population. Rouse M et al, in 2004, found a mean value for the population without binocular vision dysfunctions of 11.0 ± 8.2 (30). In 2020, González-Pérez, M et al. studied a cohort of Spanish students with mean age below that of our sample (mean age 18.6 years), and showed that the median ± interquartile range values was of 16.1 ± 28.1 (31). There are several factors that can contribute to the discrepancies in our results and those that have been previously published. The age of the population, the fact that our population was composed of men only, and their profession, could be the main ones. Nunes et al. recently published data reporting a lower CISS score in a male university population compared to the female population, attributing this difference to the male students in the study demonstrating a greater adaptive capacity (32).

Although it has been found that football players spend many hours a day using digital devices it is also true that they spend at least two hours daily outdoors training or playing. This fact can contribute to the decrease in symptomatology when compared to other populations, namely, student populations. Momeni-Moghaddam, H et al. published a paper in 2012 in which they verified a variation in the parameters of binocular vision for different body mass indexes (33). Although this study does not intend to study this relationship, it is possible to speculate whether the football players' physical constitution may influence the difference in symptoms presented by this population. This topic deserves to be investigated in detail in the future.

Regarding the time spent in high visual demand activities, the use of smartphones was the one that was mentioned by the greatest number of football players and used for more hours a day than any other device. More than 1/3 of football players spend at least 4 h a day using their smartphone. Recent publications report an increase in the number of hours spent using mobile phones in recent years. Al Shahrani et al. found that 52.5% of university students use their phones for four or more hours a day, which is higher than the average for the general population (34, 35).

The medical and technical departments of several teams reported informally that football players spent many hours using their smartphones and that there is so much concern the staff of some clubs have already taken steps to reduce the use of smartphones by football players. The concern of the clubs is not only with the visual changes that excessive use can cause but also with changes in posture and the circadian rhythm, the same concerns faced by other populations (36–38).

The existence of a direct and positive relationship between visual dysfunctions, be it a refractive error or a binocular vision, and ocular symptomatology is well documented in the literature. Recently, several papers have been published that, in addition to reporting an increase in ocular symptomatology, also report an increase in musculoskeletal symptomatology, such as an increase in neck and shoulder pain and postural changes.(6, 7, 39, 40).

In 2020, Fortes et al. published the results of an investigation where they found that the use of a smartphone for 30 min influences decision-making in football players (41). Bearing in mind that all players who completed our survey stated that they spend at least 1 h a day using their smartphone, it is imperative to study the influence of using this device not only on decision-making but on other important visual skills for playing football, such as reaction time or peripheral perception.

This survey allowed the quantification of the time spent by football players using equipment that emits blue light, namely, smartphones, and confirmed the suspicions of the medical staff of the different clubs concerning this matter. However, the survey did not highlight the time of the day electronic devices were used. In future research, this information may be useful to optimize sleep and rest monitoring strategies.

Digital eyestrain and computer vision syndrome are popularized names in the scientific and clinical community that express the relationship between the use of digital devices and the appearance of visual and ocular symptoms. The huge increase in smartphone usage has led to the publication of several studies that associate its use with a range of symptoms of ocular discomfort and asthenopia (42, 43). Some of these studies report that the symptoms caused by these types of devices are greater than those caused by the use of computers or reading printed material (44), and point out the shortening of the working distance as one of the factors for this. Golebiowski, B et al. report a decrease in the working distance from 33.8 cm to 29.5 cm when using a smartphone for 60 min (45). Long J et al. also found that smartphone users reduce their working distance as time passes. In a 60-minute experimental study, they found that the average distance of use was 30.6 ± 7.2 cm in the first 10 min and 27.8 ± 7.7 cm in the last 10 min (46).

The survey developed in this study did not provide us with data on the working distance for the different vision activities. Therefore, we cannot conclude whether what happens to other populations also applies to our population. For this reason, it could be important to develop an investigation that allows relating visual symptoms not only to the hours of use of digital devices but also to the conditions of use, namely, the distance of use of each device and the time of the day.

Current literature also points out that the use of smartphones, in addition to changes in binocular vision, has repercussions on the blink rate and tear function.(12, 45).

We also found that football players who wear glasses or contact lenses in their daily activities, including sports, exhibit a higher CISS score than those who do not use eyewear corrective items. Recently, some authors have reported a positive correlation between symptoms associated with contact lens wear and binocular vision dysfunctions (47, 48). As this study did not allow for control over the quality of contact lens adaptation, there may be a confounding mix of factors contributing to the increased symptomatology in this part of the sample.

With this research work, it was not possible to associate the results of visual symptoms with the activities carried out daily. However, it was possible to associate the appearance of symptoms with the use of a smartphone. It was found that 36.5% of football players say that the onset of symptoms or the worsening of symptoms occurs after using a smartphone.

As mentioned earlier, the medical and technical staff of football clubs have found that football players spend a lot of time in visually demanding activities, such as using a smartphone. Recent publications report the existence of visual dysfunctions in football players at percentages identical to those of the general population.

The present study has some limitations that, although reducing its robustness, do not call into question its validity. The study's primary limitation lies in its reliance on self-reported data, which can be unreliable and susceptible to biases. This subjectivity poses challenges in verifying the findings and establishing a definitive causal relationship. Future research should consider utilizing more objective data collection methods, such as wearable devices and clinical eye examinations, to provide a more robust and conclusive understanding of this relationship. The study also failed to control for crucial variables that could influence the findings, such as visual acuity, refractive error, dry eye syndrome, and other factors affecting the ocular surface. To ensure greater rigor and clarity in future research, it is imperative to carefully control for these variables, enabling a more accurate interpretation of the relationship between smartphone usage, visual dysfunctions, and athletic performance in football players. Two significant limitations of the study are that it does not directly measure the time spent on activities with high visual demand and that it uses subjective measures of athletic performance, instead of relying on specialized websites for statistical analysis of the sports performance of football players.

Future research work should be undertaken to enable a comparison between survey results and visual assessment outcomes. Additionally, objective metrics for athletic performance, such as statistical data, should be incorporated. Extending this type of investigation to other sports and athletes with different competitive levels is also advisable.

Despite its limitations, this study is a valuable contribution to our understanding of the relationship between smartphone use, visual symptoms, and athletic performance in football players. It is necessary to understand the visual habits of football players and to determine whether their daily visual activities contribute to an increase in visual symptoms and whether this can affect their sporting performance. This research work aims to contribute to the deepening of this knowledge.

## Conclusion

This study has demonstrated that professional football players engage in high visual demand tasks for a significant portion of their day, particularly on smartphones. One-third of the football players reported an association between smartphone use and the onset of ocular symptoms. The Convergence Insufficiency Symptoms Survey revealed that 6.3% of football players experienced binocular vision dysfunction symptoms. Additionally, football players with fewer ocular symptoms reported better self-perceived performance compared to those with more frequent symptoms.

## Data Availability

The raw data supporting the conclusions of this article will be made available by the authors, without undue reservation.
